# Inverse relationship between circulating sphingosine-1-phosphate and precursor species and coronary artery calcification score in type 2 diabetes

**DOI:** 10.1186/s12933-025-02624-9

**Published:** 2025-02-21

**Authors:** Wilfried Le Goff, Olivier Bourron, Clément Materne, Sophie Galier, Franck Phan, Sophie Tan-Chen, Isabelle Guillas, Agnès Hartemann, Joe-Elie Salem, Alban Redheuil, Fabienne Foufelle, Hervé Le Stunff, Eric Hajduch, Maryse Guerin

**Affiliations:** 1 INSERM, Foundation for Innovation in Cardiometabolism and Nutrition (ICAN), UMR_S1166, Sorbonne Université, 75013 Paris, France; 2 Diabetology Department, Assistance Publique-Hôpitaux de Paris (AP-HP), Hôpital Pitié-Salpêtrière, Paris, France; 3 AP-HP, INSERM, CIC-1901, Hôpital Pitié-Salpêtrière, Sorbonne Université, Paris, France; 4 Laboratoire d’Imagerie Biomédicale INSERM_1146, CNRS_7371, ICT Cardiovascular and Thoracic Imaging Unit, Assistance Publique‑Hôpitaux de Paris (AP-HP), Hôpital Pitié-Salpêtrière, Paris, France; 5 CNRS UMR 9197, Institut des Neurosciences Paris-Saclay, Université Paris-Saclay, Saclay, France; 6 Centre de Recherche Des Cordeliers, INSERM, Sorbonne Université, Paris, France

**Keywords:** Sphingosine-1-phosphate, High-density lipoprotein, Type 2 diabetes, Atherosclerosis, Cardiovascular diseases

## Abstract

**Background:**

Sphingosine 1-phosphate (S1P) is a key mediator of lipid signaling with strong immunomodulatory and anti-inflammatory effects. Circulating S1P levels including S1P in high-density lipoproteins (HDL) were demonstrated to be inversely associated with cardiovascular diseases (CVD). However, no studies are available regarding a potential implication of S1P on the risk of CVD in type 2 diabetes (T2D). The objective of this study is to determine if the increased CVD risk in T2D may involve an alteration of circulating S1P species as well as their precursors.

**Methods:**

A total of 168 and 31 patients with T2D (154 men and 45 women) with available Coronary artery calcification (CAC) score from the DIACART and CERABIAB cohorts, respectively, were included in the study. Quantification of S1P species and their precursors was carried out by LC–MS/MS in plasma and isolated HDL. CAC score was modeled as a binary variable (0/1 below or equal/above 100) using CAC < 100 for reference. S1P species or precursors were modeled as binary variables dichotomized at the median (0/1: below or equal/above the median). The relationships between S1P species and CAC score modeled as a binary variable (below or equal/above 100) was evaluated by linear regression analyses. In vitro experiments were conducted to evaluate the contribution of HDL-S1P content on anti-inflammatory properties of HDL particles.

**Results:**

Multivariate analysis revealed that plasma S1P levels, especially d18:1-S1P, and sphingosine in HDL were inversely associated with the high risk of CVD (CAC > 100) in patients with T2D. Clustering of HDL according to their concentration in S1P species and their precursors revealed that S1P-impoverished HDL is a major feature of patients with a CAC > 100. In vitro analysis of monocyte adhesion and inflammation in human umbilical vein endothelial cells as well as inflammatory phenotype of human macrophages demonstrated that low HDL-S1P exhibited impaired anti-inflammatory properties in comparison to high HDL-S1P.

**Conclusion:**

This study unraveled that circulating S1P and their precursors are biomarkers of coronary atherosclerosis in T2D, which may underlie the lower abundance of S1P and anti-inflammatory activities of HDL.

*Trial registration* ClinicalTrials.gov number, NCT02431234.

**Graphical abstract:**

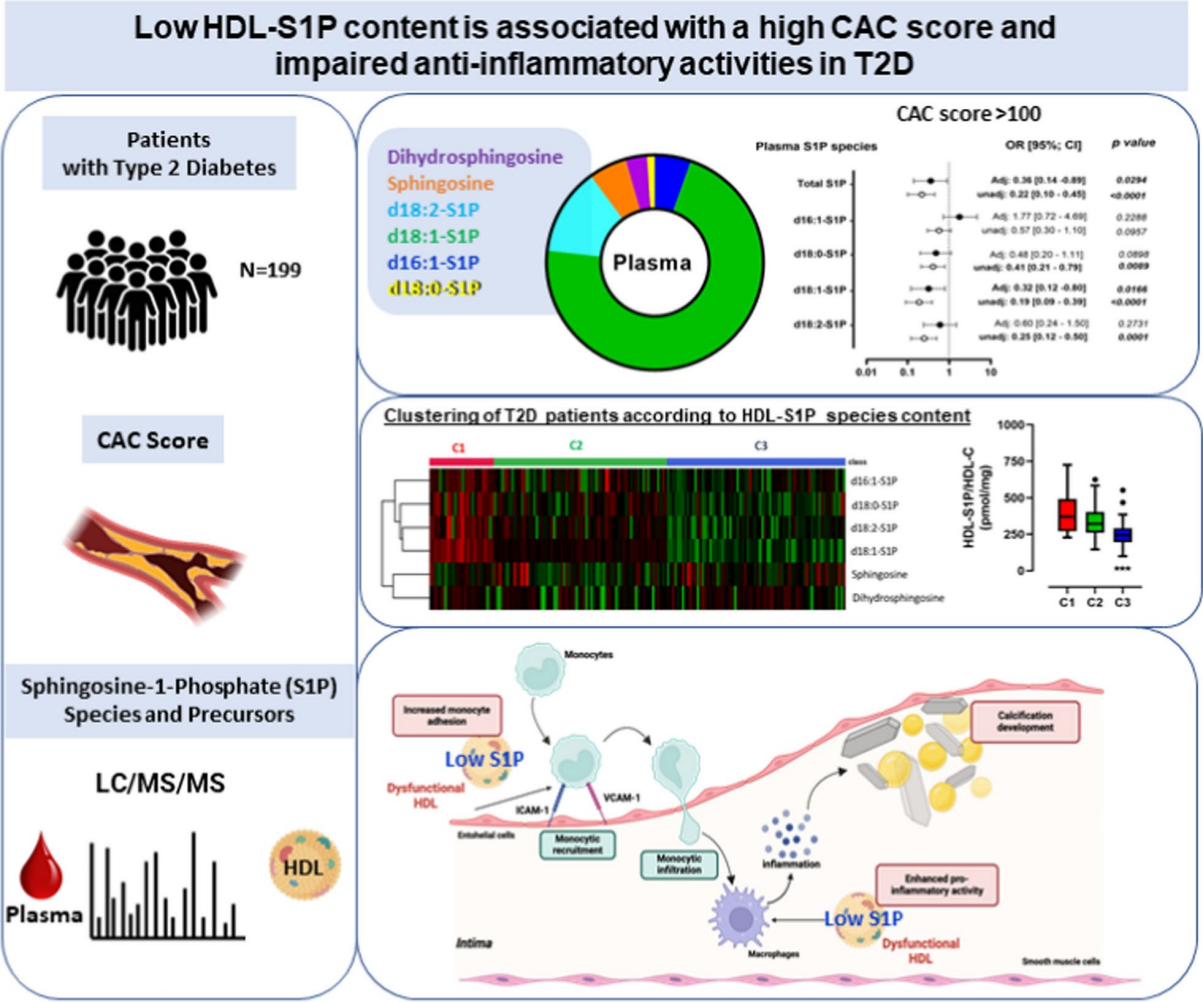

**Supplementary Information:**

The online version contains supplementary material available at 10.1186/s12933-025-02624-9.

## Introduction

Type 2 diabetes (T2D) is a major worldwide heath problem that reduces life-span [1]. Cardiovascular outcomes represent a leading cause of mortality in patients with T2D [2]. It is well established that T2D is associated with enhanced atherosclerosis progression [3]. Pathophysiology of atherosclerosis involves two major components including lipid accumulation and chronic inflammation within the arterial wall [4]. In the context of T2D, factors contributing to acceleration of atherosclerosis development include an abnormal plasma lipid profile, characterized by increased circulating levels of small dense low density lipoprotein particles together with a low high-density lipoprotein-cholesterol (HDL-C) phenotype, and an increased oxidative stress and inflammation [3, 5]. It has been demonstrated that hyperinsulinemia and hyperglycemia as observed during T2D contribute to the reduction of circulating levels of HDL-C and alteration of HDL particle functionality, both underlying the progression of atherosclerosis [6]. However, circulating levels of HDL-C neither exert nor reflect any of the potentially anti-atherogenic activities of these particles [7], suggesting that alteration of HDL functions could be related to other specific bioactive components.

HDL particles encompass numerous either proteins or lipids that constitute HDL proteome [8] and lipidome [9], which are intimately link to biological activities of HDL particles. Among more than 300 lipid components of HDL, a sphingolipid namely sphingosine-1-phosphate (S1P) is a bioactive lipid of particular interest in the regulation of cardiovascular diseases [10]. S1P is synthesized primarily in platelets, erythrocytes, and endothelial cells and is transported bound to HDL and/or albumin [11–13]. In the plasma, S1P is mainly transported by HDL (55%), the remaining being associated to other lipoproteins (10%) or to serum albumin (35%) [14, 15]. Interestingly, it has been shown that some of the atheroprotective actions of HDL are mediated by S1P [13, 16]. Examples of S1P-dependent HDL functions include nitric oxide (NO) production by endothelial NO synthase and subsequent arterial vasodilation [17], or inhibition of TNFα-induced adhesion molecule expression in endothelial cells [18]. In addition, it has been demonstrated that the HDL-associated apoM–S1P complex mediates vasoprotective actions on the endothelium [19]. A number of studies have also established a link between plasma S1P or HDL-S1P concentrations and the occurrence of cardiovascular events such as incidence of myocardial infarction, incidence of intra-stent restenosis and angiographic severity of coronary artery disease [13] or mortality specifically in African Americans with T2D [20].

**Fig. 1:**
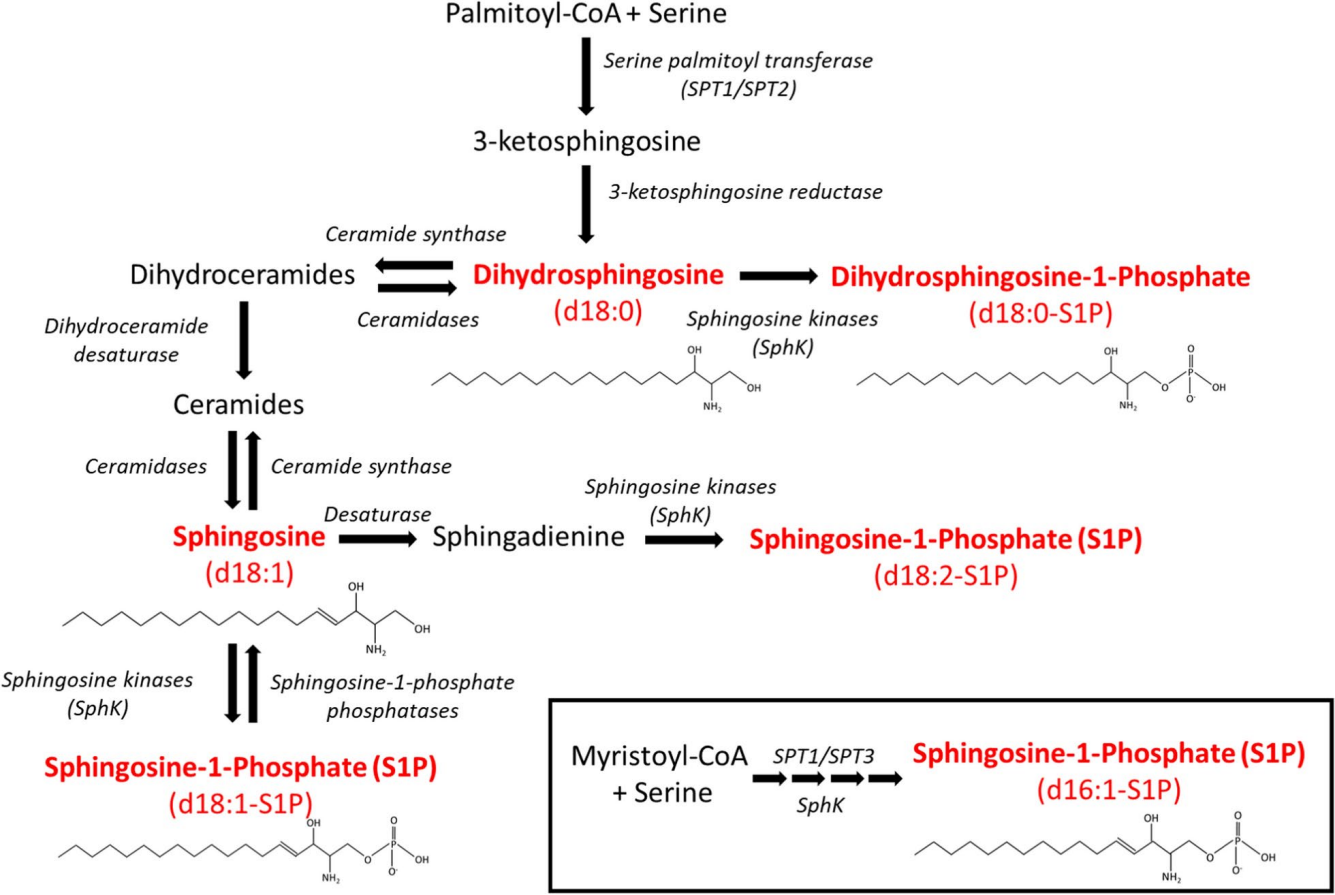
Biosynthesis pathways of sphingosine-1-phosphate species. Major biosynthesis pathways of Sphingosine-1-Phosphate species (d16:1-S1P, d18:0-S1P, d18:1-S1P and d18:2-S1P) and their precursors (dihydrosphingosine and sphingosine) (in red). Dihydrosphingosine (Sphinganine); S1P, Sphingosine-1-Phosphate; SphK, sphingosine kinase; SPT, Serine palmitoyl transferase

In most studies concerning S1P and its function in cardiovascular diseases, S1P refers to the predominant form of phosphorylated sphingoid bases and is also called d18:1-S1P. The d18:1-S1P is synthetized from the phosphorylation by two sphingosine kinase (SphK), namely SphK1 and SphK2, of sphingosine which is produced by de-acylation of ceramide by ceramidase [21] (Fig. 1). The second abundant S1P specie, d18:0-S1P, corresponds to the saturated form of S1P, which is produced by the phosphorylation of dihydrosphingosine (sphinganine) by SphKs [22]. Dihydrosphingosine is produced through the de novo ceramide synthesis pathway initiated by serine palmitoyl transferase (SPT) activity which generates 3-ketosphingosine, which is rapidly reduced to dihydrosphingosine via 3-ketosphingosine reductase [23] (Fig. 1). While the combination of SPT1 and SPT2 isoforms primarily form d18 sphingoid bases, it has been shown that the SPT1/SPT3 complex produces a broader spectrum of sphingoid bases including d16-sphingoid bases [24] (Fig. 1). Interestingly, d16:1-S1P could be produced by SphKs and be found in plasma [25]. A last sphingoid base is sphingadienine (d18:2) which is likely generated by the desaturation of sphingosine through an yet unknown enzyme [26] (Fig. 1). It is also metabolized by phosphorylation to sphingosine-1-phosphate (d18:2-S1P) and detected in both cell and plasma [26] (Fig. 1).

It is interesting to note the emerging role of different S1P species in cardiovascular disease. Both plasma S1P and HDL-S1P were associated with the coronary artery disease [27, 28]. Circulating d18:0-S1P was also correlated with ischemic heart disease [29] and although d18:2-S1P have not been much studied in plasma, they were found to be inversely linked to the risk of major cardiovascular events [29]. Moreover, and independently of obesity and T2D, some atypical S1P species were associated with the risk of major cardiovascular events [30].

Despite the respective association of circulating level of total HDL-S1P and cardiovascular diseases, studies on this topic in type 2 diabetes are rare. Previous studies reported that both ApoM- and HDL-associated d18:1-S1P were decreased in patients with T2D [31–33], however none evaluated the association between circulating concentrations of S1P species and their precursors and the risk of cardiovascular diseases in patients with T2D.

To this aim, we carried out a comprehensive quantification of S1P species and their precursors in plasma and HDL from two very well phenotyped cohort of patients with T2D by tandem mass spectrometry. We also evaluated the coronary artery calcification (CAC) score, a well-recognised surrogate marker for the existence and severity of coronary atherosclerosis [34]. Using a high dimensional statistical analysis, we evaluated the relationship between the presence of those sphingosine base phosphate species in plasma and HDL from T2D patients and their CAC score.

We unravelled that the specific composition of plasma and HDL with S1P and their precursors allow to stratify the risk of cardiovascular events in patients with T2D. Finally, we demonstrated that the relationship between predicted cardiovascular risk and HDL-S1P levels in patients with T2D is linked to the anti-inflammatory properties of HDL.

## Methods

### Study population and blood samples

The study population included 199 patients with T2D from the DIACART and the CERADIAB Studies [35, 36].

DIACART is a prospective observational study including 198 patients with T2D from the Diabetes and Cardiology Departments of the Pitié-Salpêtrière Hospital (Paris, France) to assessing the factors associated with lower limb arterial calcification [35]. Inclusion criteria were T2D with at least 1 of the following risk factors: coronary artery disease (CAD) or peripheral arterial occlusive disease or age > 50 years for men and > 60 years for women. Exclusion criteria were an estimated glomerular filtration rate < 30 mL/min, a history of lower limb angioplasty and/or bypass, immunodeficiency, type 1 diabetes. All patients with infectious disease at inclusion with clinical and/or biological signs of inflammation were excluded. During follow-up of the study, 18 patients were lost and 11 patients died before the end of the study. Thus, at the end of the study, sphingolipid concentration was measured in 168 patients. The study was approved by the local ethics committee and registered in ClinicalTrials.gov (NCT02431234). Participants gave their written informed consent of participation prior to inclusion.

CERADIAB is a cross-sectional study involving 90 patients with T2D attending a day hospital in the Diabetes Department of the Pitié-Salpêtrière Hospital (Paris, France) to assess the plasma concentrations of sphingolipids. Inclusion criteria was: Patients with T2D. Exclusion criteria were: Diabetes without overweight, diabetes with autoimmune markers of type 1 Diabetes, post-transplantation diabetes mellitus, Maturity Onset Diabetes of the Young (MODY), diabetes diagnosed before 35 years old, patients taking medications such as glucocorticoids, HIV medications and atypical antipsychotics, immunosuppressive therapy, patients with type 1 and type 2A dyslipidemia, non-metabolic hepatopathy, cirrhosis, severe renal failure defined by an estimated glomerular filtration rate by the modification of diet in renal disease equation (MDRD) < 30 ml/min. Of the 90 patients included, coronary artery calcification score was available in only 31 patients. The study was approved by the local ethics committee.

Blood samples were obtained after overnight fasting by venipuncture into EDTA-containing Vacutainer tubes. Plasma was rapidly separated by low speed centrifugation and stored at -80 °C until analysis.

### Evaluation of atherosclerotic burden

Coronary artery calcification (CAC) measurements were performed for each patient by using a multi-detector CT scan (Definition Flash, Siemens, Erlangen, Germany) as previously described [37]. CAC was quantified according the Agatston scoring method [38]. The presence of CAC was evaluated using semi-automated calcium scoring software SyngoVia (Siemens) over the entire epicardial coronary tree. The total calcium score was calculated by summing CAC scores from the left main, left anterior descending, left circumflex, and right coronary arteries.

### Isolation of HDL

High-density lipoproteins (HDL) (d: 1.063–1.21 g/ml) were prepared from plasma by sequential ultracentrifugation using a Beckman Optima Max-TL centrifuge. Briefly, the density of plasma was increased to 1.063 g/ml. Following a first centrifugation at 120,000 rpm at 15 °C for 3 h, the d < 1.063 g/ml fraction was removed, and the density of the fraction d > 1.063 g/ml was increased to 1.21 g/ml. The HDL fraction was subsequently obtained following a second centrifugation at 120,000 rpm at 15 °C for 5 h. After isolation, lipoprotein fractions were analyzed for their lipid and protein content. Lipids and protein analyses were performed with an Autoanalyzer (Konelab 20). Commercial reagent kits from Diasys were used for total cholesterol (TC), free cholesterol (FC) and Phospholipids (PL). Triglycerides (TG) were quantified with reagent kits from ThermoElectron. Bicinchoninic acid assay reagent (Pierce) was used for total protein quantification. High-density lipoproteins (HDL) fraction was dialyzed exhaustively against PBS before use for lipidomic and functional analyses.

### S1P quantification

S1P content in plasma or in isolated HDL was quantified by liquid chromatography coupled with tandem mass spectrometry (LC–MS/MS) as previously described [39, 40]. Briefly, 50 µL of plasma or the dialyzed HDL fraction were fortified with 900 nmol/L d17:1-S1P (#860,641, Avanti Polar Lipids, Alabaster, USA) as internal standard. The samples were twice extracted with 2 mL of isopropanol:water:ethyl acetate (30:10:60 v/v) by vortexing for 30 min and centrifuging for 5 min at 1100 g. The upper organic phase was evaporated with a SpeedVac system. The dried residues were reconstituted with methanol, and were analyzed on a LC–MS/MS system (6460 mass spectrometer, Agilent Technologies, Santa Clara, USA) using a Poroshell 120 EC-C8 column (#685,775–906, Agilent). S1P and internal standard were detected using multiple reaction monitoring (m/z 380 → 264 and 366 → 250, respectively), and were quantified using a seven-point calibration curve with d18:1-S1P as external standard (#860,492, Avanti Polar Lipids).

### Evaluation of anti-inflammatory activities of HDL particles

Experiments were performed using the THP-1 human monocytic cell line (ATCC) or human umbilical vein endothelial cells (hUVEC) (Promocell) subcultured for a maximum of 5 passages in complete endothelial cell growth medium (Promocell). THP-1 cells were differentiated into macrophage-like cells after treatment with 50 ng/mL phorbol 12-myristate 13-acetate (PMA, # P8139, Sigma) for 48 h. Macrophages were treated for 4 h with serum-free RPMI medium in the presence or absence of 50 µg protein/ml of HDL. After incubation, cells were wash with PBS and treated for a further 4 h with serum-free RPMI containing 100 ng/ml of LPS (#L2630, Sigma) or not. Confluent hUVEC cells were treated for 2 h with serum-free endothelial cell medium (Promocell) in the presence or absence of 50 µg protein/ml of HDL and further stimulated with 20 ng/ml of TNFα for an additional 3 h.

### RT-qPCR analysis

Total RNA was extracted from cells using the NucleoSpin RNA II kit (Macherey–Nagel, Düren, Germany) according to the manufacturer’s instructions. Both reverse transcription and real-time quantitative PCR were performed using a LightCycler LC480 (Roche, Basel, Switzerland) using specific primers (Supplemental Table S1) as previously described [41]. mRNA levels were normalized to those of human non-POU domain–containing, octamer-binding housekeeping gene (*NONO*), human α-tubulin (*TUBA*) and human heat shock protein 90 kDa α (cytosolic) class B member 1 (*HSP90AB1*). Data were expressed as a fold change in mRNA expression relative to control values.

### Evaluation of monocytes adhesion on endothelial cells

Experiments were conducted as previously described [42] using Human umbilical vein endothelial cells (hUVEC) and human THP-1 monocytic cells. Briefly, when reached 80% of confluency, hUVEC were treated for 2 h with serum-free endothelial cell medium in the presence or absence of 50 µg protein/ml of HDL. Cells were then stimulated with 20 ng/ml of human TNFα (#SRP3177, Sigma) for an additional 3 h. In parallel, Monocytic THP-1 cells were incubated with calcein-AM Dye (#C3100MP, Thermofisher Scientific) for 15 min, then washed three times with serum-free RPMI medium (Supplemental Tables S2 and S3). Labeled THP-1 were then incubated with hUVEC following HDL and 20 ng/mL human TNFα (#SRP3177, Sigma) treatment for 30 min to allow monocyte adhesion. Non-adherent THP-1 cells were removed by washing cells 8 times with PBS and THP-1 cells bound to hUVEC were detached by trypsin–EDTA (#T4049, Sigma) before analysis by flow cytometry.

### Flow cytometry analysis

Cells were incubated with a fixable viability dye (eF520, #65–0867, eBiosciences) for 15 min, blocked with an FcR bocking reagent (#130–059-901, Miltenyi Biotech) for 20 min and stained for surface markers (antibody against ICAM-1/CD54-APC (#559,771, BD-Biosciences), VCAM-1/CD106-PE (#555,647, BD Biosciences), dilution 1/5). Cells were then fixed with FoxP3 Staining Buffer (#00–5521-00, eBioscience) and acquisition was performed on a LSR Fortessa Analyzer (BD Biosciences) (Supplemental Table S2). Details for flow cytometry acquisition are presented in Supplemental Figures S1 and S2.

### Statistical analyses

Quantitative data were expressed as mean ± SD or median (25th–75th percentile). Categorical variables were given as n (%) of patients. Statistical analyses were performed using the R statistical software computer program version Ri386 4.1.3 (R Foundation for statistical computing). Variables were tested for normal distribution using Kolmogorov–Smirnov test. Comparisons between 2 groups of subjects were performed using unpaired t-test was used for normally distributed parameters, whereas the Mann–Whitney test was used for skewed data. Quantitative variables presented as proportions were compared using the Chi square test CAC score was modeled as a binary variable (0/1 below or equal/above 100) using CAC < 100 for reference. S1P species or precursors were modeled as binary variables dichotomized at the median (0/1: below or equal/above the median). Univariate and multivariate linear regression analyses (generalized linear model) were used to determine relationships between variables and CAC score. Adjusted model included variables significantly associated with coronary calcium score in univariate analyses (Supplemental Table S4) as follows: age, sex, low-density lipoprotein-cholesterol (LDL-C), HTA, current smoking, CKD, status with regard to use of statins, dual antiplatelet therapy, angiotensin converting enzyme inhibitors/angiotensin II receptor blocker and beta blockers. Distribution of HDL-S1P species and precursors among patients were analyzed by Ward’s Hierarchical Clustering and visualized with a heatmap by using MetaboAnalyst 4.0 (Xia Lab at McGill University, Montreal, QC, Canada). Multiple comparison tests were performed using one-way analysis of variance (ANOVA) with post-hoc test. Results were considered statistically significant at p < 0.05.

## Results

### Clinical and biochemical characteristics of the cohort of patients with type 2 diabetes

Major clinical and biochemical characteristics of the whole study population are shown in **(**Table 1**).** Patients with T2D (n = 199) involved in the present study were predominantly men, active or former smokers with hypertension. Most of patients were treated with statins (83.4%), antiplatelet therapies (71.3%), β-blockers (54.4%) and inhibitors of the renin-angiotensin system (angiotensin receptors blockers (ARB) and angiotensin converting enzyme (ACE) inhibitors) (79.3%). Antidiabetic therapies predominantly involved use of metformin, insulin and sulfonylurea (respectively 79.9%, 48.7% and 45.2%). No significant difference in mean fasting blood glucose, HbA1c, diabetes duration or in BMI was observed between the two subgroups of patients with T2D stratified according to CAC score below or above 100. In addition, a significant reduction in fasting total cholesterol and LDL-C levels was observed in patients with CAC score > 100 as compared to those with a CAC score < 100. In good agreement with this latter observation, patients receiving a statin therapy predominate among patients exhibiting a CAC score > 100 (66.7% vs 89.2%, p = 0.0002). However, fasting HDL-C levels were not significantly different between the two subgroups (1.19 ± 0.47 vs 1.11 ± 0.39, p = 0.1786). Equally, a higher proportion of those patients were receiving therapies for secondary prevention including antiplatelet, renin-angiotensin system inhibitors or beta blockers.

**Table 1:** Clinical and biological characteristic of the study population according the degree of Coronary Artery Calcification score

Variables	Patients with Type 2 diabetes			*p* value
	All subjects (n = 199)	CAC < 100 (n = 51)	CAC > 100 (n = 148)	
CAC score	495 (89.8– 1470)	2.6 (0– 29.8)	926 (355.3– 1709)	< 0.0001
Age, year	65.4 ± 9.0	60.2 ± 9.2	67.2 ± 8.3	< 0.0001
Diabetes duration, years	16.6 ± 10.4	14.6 ± 10.7	17.3 ± 10.2	0.0971
Coronaropathy, n (%)	118 (59.3)	11 (21.6)	107 (72.3)	< 0.0001
Retinopathy, n (%)	49 (24.6)	10 (19.6)	39 (26.3)	0.3350
Neuropathy, n (%)	64 (32.2)	13 (25.5)	51 (35.2)	0.2047
Male Gender, n (%)	154 (77.4)	34 (66.7)	120 (81.1)	0.0338
BMI, kg/m^2^	29.1 ± 5.2	28.6 ± 4.8	29.2 ± 5.3	0.4991
HTA, n (%)	160 (84.5)	32 (62.7)	128 (86.5)	0.0002
SBP, mmHg	134.7 ± 17.1	131.4 ± 13.9	135.8 ± 18.0	0.1091
DBP, mmHg	76.0 ± 11.1	76.0 ± 7.4	76.0 ± 12.2	0.9677
Smoking habit, n (%)	125 (62.8)	25 (49.0)	100 (67.6)	0.0181
FBG, mmol/l	9.06 ± 3.51	9.57 ± 4.61	8.88 ± 3.05	0.2251
HbA1c, %	7.77 ± 1.21	7.95 ± 1.30	7.71 ± 1.70	0.2196
eGFR MDRD, ml/min	79.6 ± 24.0	86.0 ± 25.0	77.4 ± 23.3	0.0288
CKD, n (%)	108 (54.3)	21 (41.2)	87 (58.8)	0.0295
Triglycerides, mmol	1.49 (1.02–2.17)	1.33 (0.96–2.05)	1.57 (1.03–2.25)	0.2100
Total cholesterol, mmol/L	4.09 ± 0.98	4.40 ± 1.16	3.98 ± 0.91	0.0071
LDL-C, mmol/L	2.12 ± 0.80	2.48 ± 0.98	1.73 ± 0.70	< 0.0001
HDL-C, mmol/L	1.14 ± 0.41	1.19 ± 0.47	1.11 ± 0.39	0.1786
Metformin, n (%)	159 (79.9)	41 (80.4)	118 (79.7)	0.9189
Sulfonylurea, n (%)	90 (45.2)	22 (43.1)	68 (46.0)	0.7282
DPP4 Inhibitor, n (%)	55 (27.6)	14 (27.5)	41 (27.7)	0.9723
αglucosidase inhibitor, n (%)	2 (1.0)	1 (2.0)	1 (0.7)	0.4275
Glinide, n (%)	6 (3.0)	3 (5.9)	3 (2.0)	0.1650
GLP1 receptor agonist, n (%)	12 (6.0)	6 (11.8)	6 (4.0)	0.0461
Insulin, n (%)	97 (48.7)	23 (45.1)	74 (50.0)	0.5459
Statin, n (%)	166 (83.4)	34 (66.7)	132 (89.2)	0.0002
Ezetimibe, n (%)	23 (11.5)	4 (7.8)	19 (12.8)	0.3360
Fibrates, n (%)	9 (4.5)	2 (3.9)	7 (4.7)	0.8107
Antiplatelet, n (%)	142 (71.3)	17 (33.3)	125 (84.5)	< 0.0001
ARB and ACE inhibitors	147 (79.3)	28 (54.9)	119 (80.4)	0.0004
Beta Blockers, n (%)	108 (54.3)	13 (25.5)	95 (64.2)	< 0.0001

Values are mean ± SD, median (interquartile range) or number (percentage). *ACE* Angiotensin converting enzyme, *ARB* Angiotensin receptor blockers, *BMI* Body Mass Index, *CAC* Coronary artery calcification, *CKD* Chronic kidney disease, *DBP* Diastolic blood pressure, *eGFR-MDRD* Estimated glomerular filtration rate according to the Modification of Diet in Renal Disease equation, *FBG* Fasting blood glucose, *HDL* High-density lipoprotein-cholesterol, *HTA* Hypertension, *LDL-C* Low-density lipoprotein-cholesterol, *SBP* Systolic blood pressure. Smoking habit represents active and ex-smokers

### Circulating levels of S1P species and precursors according to the coronary artery calcification of patients with type 2 diabetes

As shown in Table 2**,** circulating levels of S1P species and precursors (dihydrosphingosine and sphingosine) determined in either whole plasma or isolated HDL showed large variations between individuals. The inter-individual variabilities expressed as the 95th percentile ratio to the 5th percentile of S1P species detected in isolated HDL were higher than those observed in plasma. To note, the d18:1-S1P represented the most abundant S1P species accounting for at least 80% of total S1P in both plasma and HDL particles (Supplemental Figure S3). Stratification of patients according to the degree of CAC revealed reduced plasma levels of all S1P species in patients with CAC > 100 as compared to those with CAC < 100 (Table 2). Interestingly, approximately 1/3 of plasma S1P species was retrieve in isolated HDL, thus indicating that 30% or less of circulating S1P was associated to HDL particles in patients with T2D. Those proportion appear even lower in subgroup of patients exhibiting a CAC score > 100 **(**Table 2**)**.

**Table 2:** Quantification of circulating levels of S1P species and precursors according to Coronary Artery Calcification score

S1P species and precursors	CAC < 100			CAC > 100					Fold change from CAC < 100
	Median (Q1–Q3)	Mini	Maxi	Interindividual variability	Median (Q1–Q3)	Mini	Maxi	Interindividual variability	
*Concentration in plasma (pmol/ml)*									
Total S1P	591.6 (513.9–797.6)	354.7	1143	2.93	493.4 (437.1–580.4)	276.8	1230	2.55	− 1.20***
d16:1-S1P	33.4 (25.0–42.9)	15.2	74.1	3.79	29.5 (25.3–35.5)	12.5	80.5	2.66	− 1.13*
d18:0-S1P	8.17 (6.52–11.3)	2.81	17.1	2.96	6.86 (5.82–8.34)	3.26	20.8	3.24	− 1.19**
d18:1-S1P	483.7 (420.1–641.9)	265.9	927.8	2.93	392.3 (342.2–460.9)	178.8	1023	2.58	− 1.23***
d18:2-S1P	85.4 (71.2–108.8)	46.0	161.6	2.9	73.8 (64.3–85.7)	32.5	150.7	2.3	− 1.16***
*Precursors*									
Dihydrosphingosine	19.0 (17.8–24.5)	15.8	32.7	1.91	17.7 (17.0–18.7)	14.8	28.2	1.42	− 1.07***
Sphingosine	27.3 (21.4–37.5)	9.6	95.0	6.42	25.3 (18.1–39.0)	9.7	182.9	8.91	− 1.08
*Concentrations in isolated HDL particles (pmol/ml)*									
Total S1P	199.4 (131.2–262.0)	60.7	335.1	4.11	129.0 (103.64–150.6)	31.4	542.7	3.43	− 1.55***
d16:1 S1P	17.7 (10.3–24.5)	4.67	37.9	6.14	11.1 (9.10–14.3)	4.14	43.1	4.60	− 1.59***
d18:0-S1P	0.96 (0.60–1.41)	0.24	1.93	5.79	0.78 (0.56–1.02)	0.19	1.98	4.03	− 1.23***
d18:1 S1P	149.0 (107.1–195.4)	49.3	248.1	4.02	102.4 (80.3–117.0)	22.8	426.0	3.58	− 1.46***
d18:2 S1P	27.7 (16.2–41.1)	6.73	64.1	6.81	15.2 (12.1–18.4)	2.58	73.6	5.30	− 1.82***
*Precursors*									
Dihydrosphingosine	26.7 (25.1–28.2)	20.7	31.7	1.48	28.4 (26.2–31.2)	19.0	37.7	1.58	+ 1.06***
Sphingosine	23.6 (14.4–43.3)	7.48	87.5	8.79	15.7 (11.4-21.7)	6.01	98.1	4.59	-1.50***

Results expressed as median (Q1–Q3). Interindividual variability expressed as the ratio of 95th percentile/5th percentile

^*^p < 0.05; **p < 0.005; ***p < 0.0005. *CAC* Coronary artery calcification, *S1P* Sphingosine-1-phosphate

Similar levels of plasma S1P precursors (sphingosine and dihydrosphingosine) were observed in both plasma and isolated HDL particles, indicating that plasma S1P precursors were primarily associated with circulating HDL particles (Table 2, Supplemental Figure S3). Interestingly, sphingosine content was significantly lower in HDL but not in plasma from patients with T2D exhibiting a CAC score > 100 while dihydrosphingosine content was significantly reduced in plasma but increased in HDL of these patients (Table 2).

Taken together, these findings revealed an overall impoverishment of circulating S1P species and precursors in T2D patients with a high risk to develop atherosclerosis.

### Sphingosine-1-phosphate species and cholesterol content are correlated in high-density lipoproteins from patients with type 2 diabetes

**Fig. 2:**
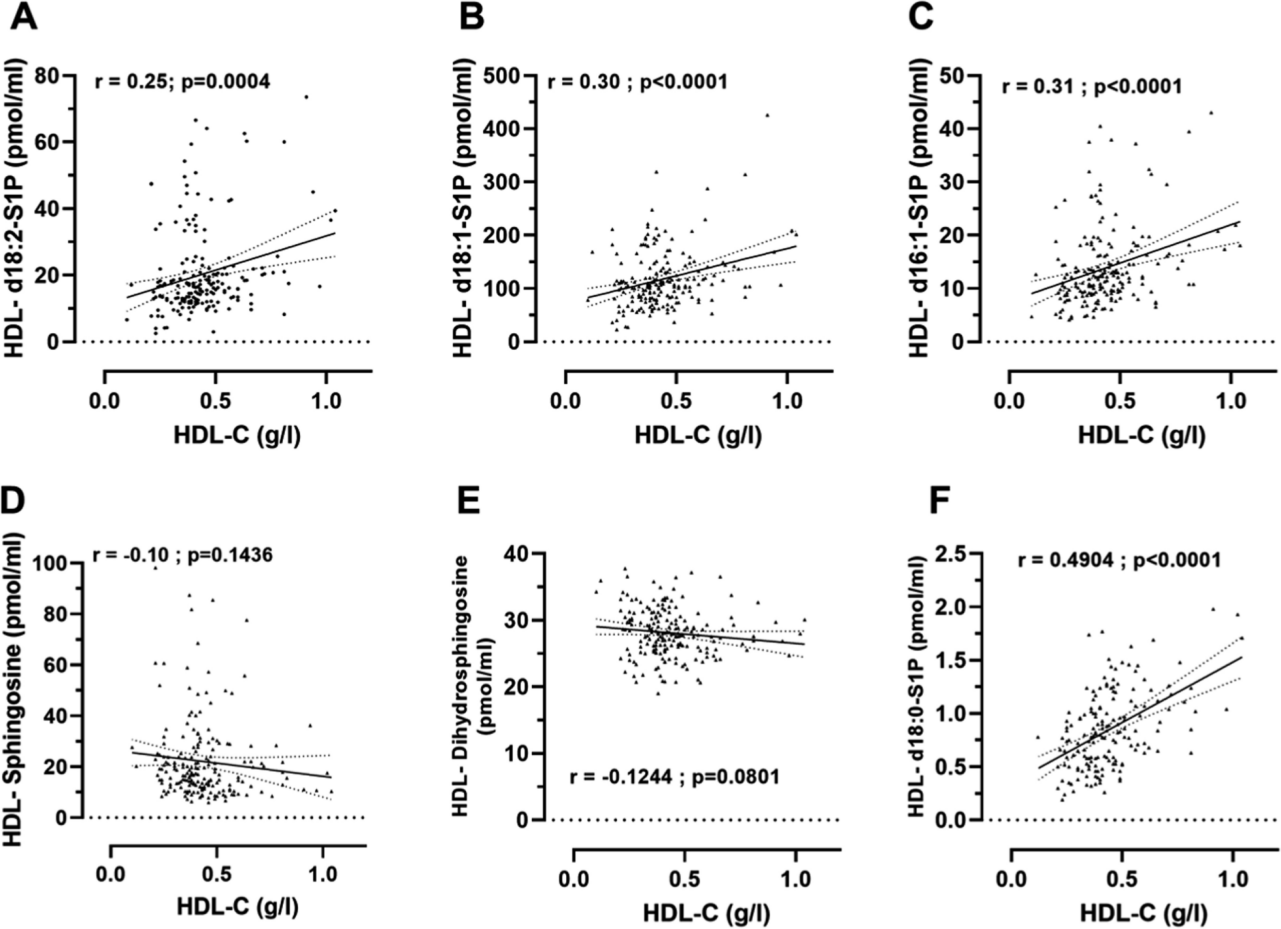
Correlation between sphingosine-1-phosphate and precursors species with cholesterol in high-density lipoproteins. Correlation between HDL S1P species, d18:2-S1P panel **A**, d18:1-S1P panel **B**, d16:1-S1P panel **C**, d18:0-S1P (panel F) or precursor, sphingosine panel **D**, dihydrosphingosine panel **E** and plasma HDL-cholesterol levels in patients with type 2 diabetes (n = 199). r indicates pearson correlation coefficient, dotted line indicate 95% confidence interval

High-density lipoproteins (HDL) cannot be considered as a unique family of particles and are classically ranged according to their density and size with large HDL being enriched in cholesterol in comparison to small HDL [9]. In an attempt to determine whether S1P species and precursors preferentially associate either with large cholesterol-rich or small cholesterol-poor HDL particles, we analyzed the relationship between S1P species and precursors levels with cholesterol levels in plasma HDL (Fig. 2). We observed that HDL-S1P species (d16:1-, d18:0-, d18:1 and d18:2-S1P) significantly correlated with HDL-C levels whereas HDL-S1P precursors (dihydrosphingosine and sphingosine) did not, indicating that S1P species were preferentially associated with large HDL particles and suggesting that S1P precursors were preferentially found in small HDL particles.

### Relationship between circulating levels of S1P species or precursors and the risk of atherosclerosis in patients with type 2 diabetes

**Fig. 3:**
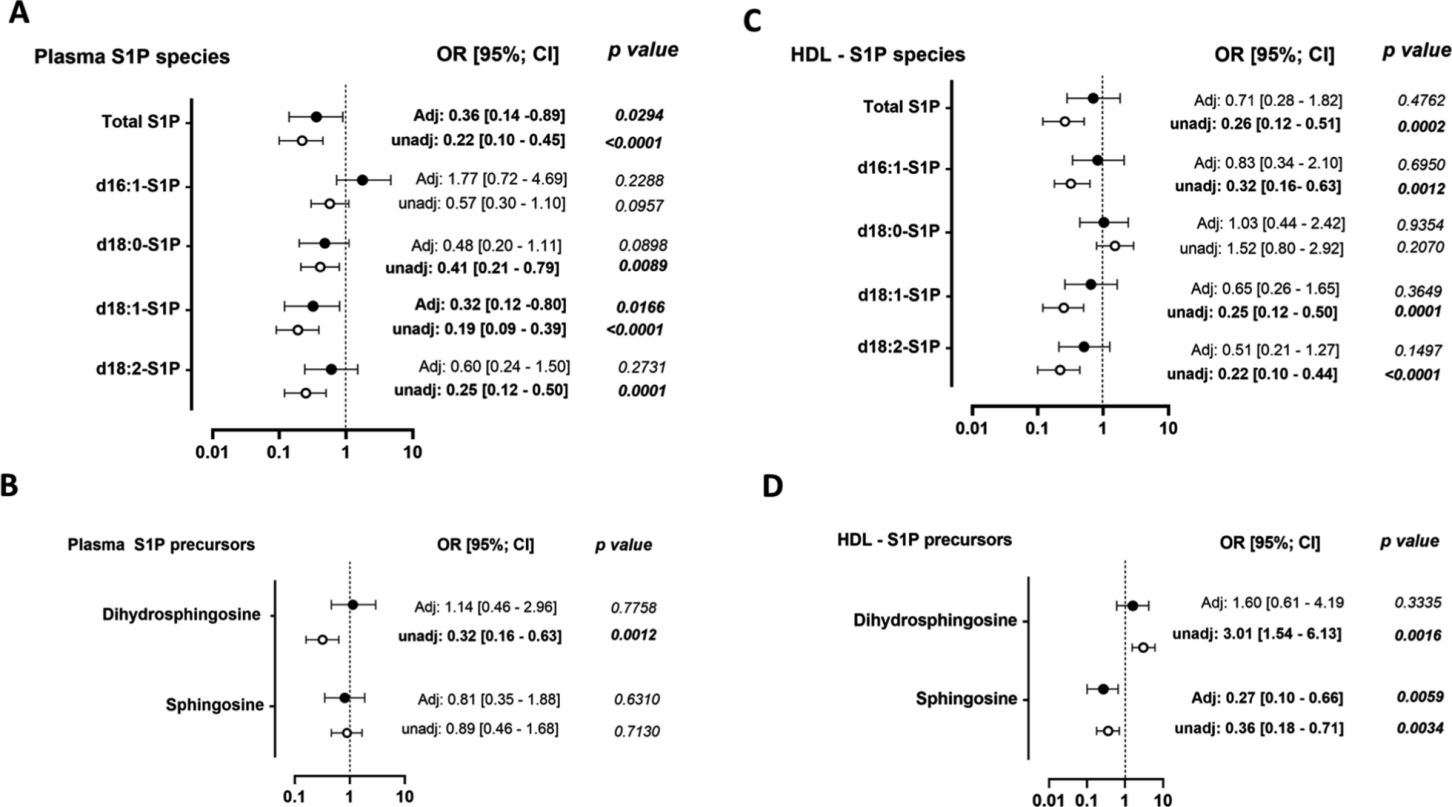
Circulating sphingosine-1-phosphate and precursor species and risk to develop a high coronary artery calcification score. Relationship between plasma levels of S1P species panel **A** and precursors panel **B** with coronary artery calcification score, CAC > 100. Relationship between plasma levels of HDL-S1P species panel **C** and precursors panel **D** with CAC > 100. Adjusted model included independent variables associated with coronary calcium score including age, sex, LDL-cholesterol, CKD, status with regard to use of statins, dual antiplatelet therapy, angiotensin converting enzyme inhibitors/angiotensin II receptor blocker and beta blockers. OR, odd ratio; CI, confidence interval. n = 199 T2D patients

To further evaluate whether low circulating levels of S1P species and precursors might contribute to the development of atherosclerosis in T2D, we determined the relationship between levels of S1P species and precursors in either plasma or HDL with the degree of CAC score (< 100 and > 100). As shown in Fig. 3, multivariate analyses revealed significant inverse associations between plasma levels of total S1P and the major d18:1-S1P specie (Fig. 3A) with CAC > 100, whereas those of S1P precursors were not (Fig. 3B). In contrast, HDL-S1P species levels were not significantly associated with the presence of a CAC score > 100 (Fig. 3C) which may be due to the low abundance of S1P species in HDL fraction (30%, Table 2). By contrast, HDL-sphingosine levels, a S1P precursor mostly associated with HDL particles, showed an inverse relationship with CAC > 100 in both univariate and multivariate analyses (Fig. 3D).

To conclude, plasma levels of S1P and HDL content of S1P precursors species in patients with T2D were inversely associated with a high risk of atherosclerosis development.

### Low content of sphingosine-1-phosphate species in high-density lipoproteins is associated with a high coronary artery calcification score

**Fig. 4:**
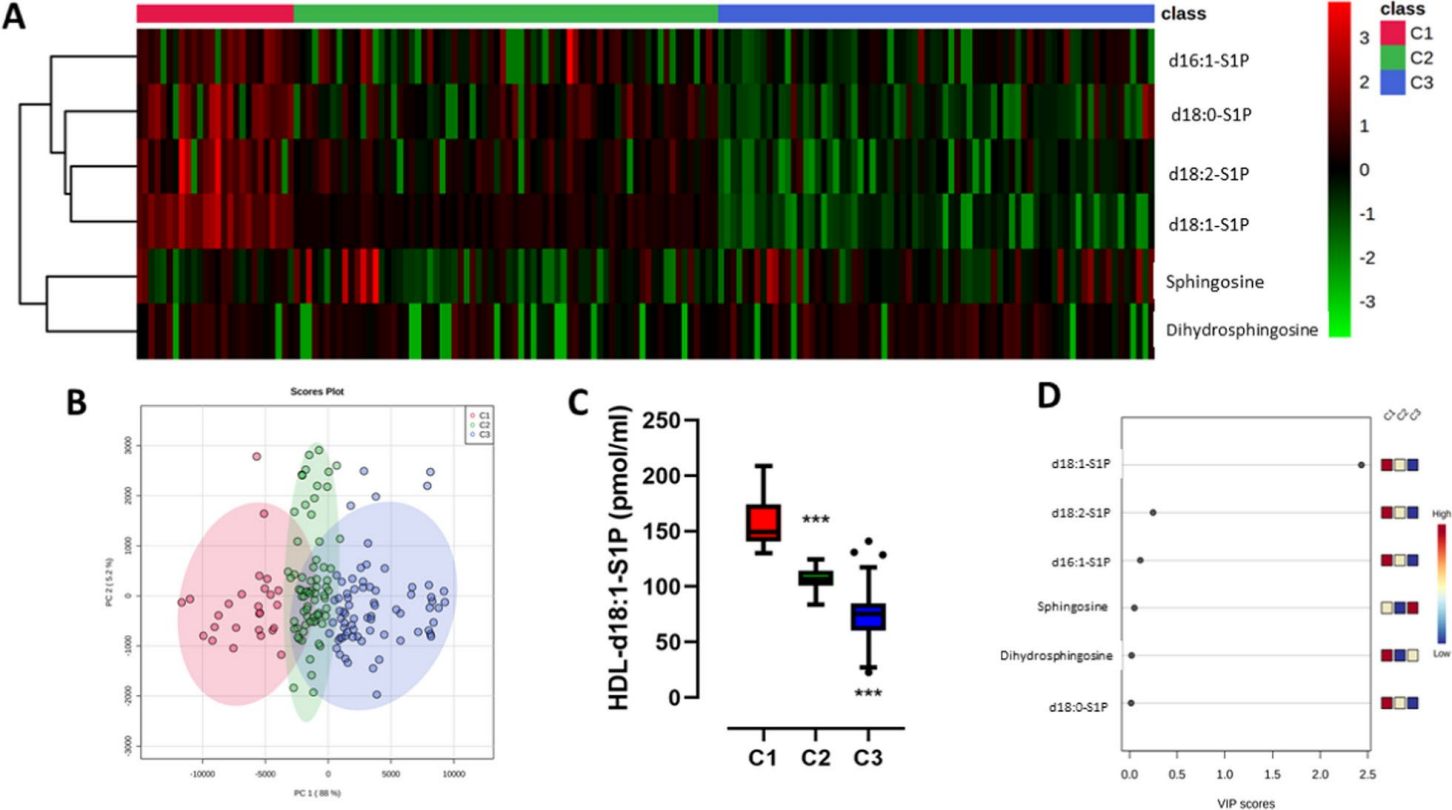
Clustering of high-density lipoproteins according to the amount of sphingosine-1-phosphate and precursor species. Hierarchical cluster of type 2 diabetic patients based on HDL-S1P species and precursors levels. Three clusters were identified and referred to C1 (n = 26, red), C2 (n = 70, green) and C3 (n = 72, blue) panel **A**. PCA score panel **B**. Box plot of HDL-d18:1-S1P levels according to clusters panel **C**. Variable importance in projection (VIP) score plot panel **D**. ***p < 0.0005 Cluster 3 vs Cluster 1

Given the individual variability of circulating levels of S1P species and precursors associated with HDL particles, we carried out an unsupervised clustering approach using Ward’s Hierarchical Clustering to further investigate the importance of these sphingolipid species in HDL from patients with T2D and a high risk of CAC score. By combining both HDL-S1P species and precursors levels of patients with T2D (n = 168), we identified three clusters referred to C1, C2 and C3 accounting for 26, 70 and 72 patients, respectively (Fig. 4A). The principal component analysis (PCA) score plot showed that the three independent clusters were well separated from each other, thus indicating distinct circulating levels of HDL-S1P species and precursors between subgroups of subjects (Fig. 4B). Individuals belonging to cluster C1 were characterized by highest concentrations of each of HDL-S1P species, whereas those from cluster C3 exhibited lowest levels, as shown for HDL-d18:1-S1P levels across clusters (Fig. 4C), which was identified as the most important feature contributing to the classification of patients (Fig. 4D).

**Fig. 5:**
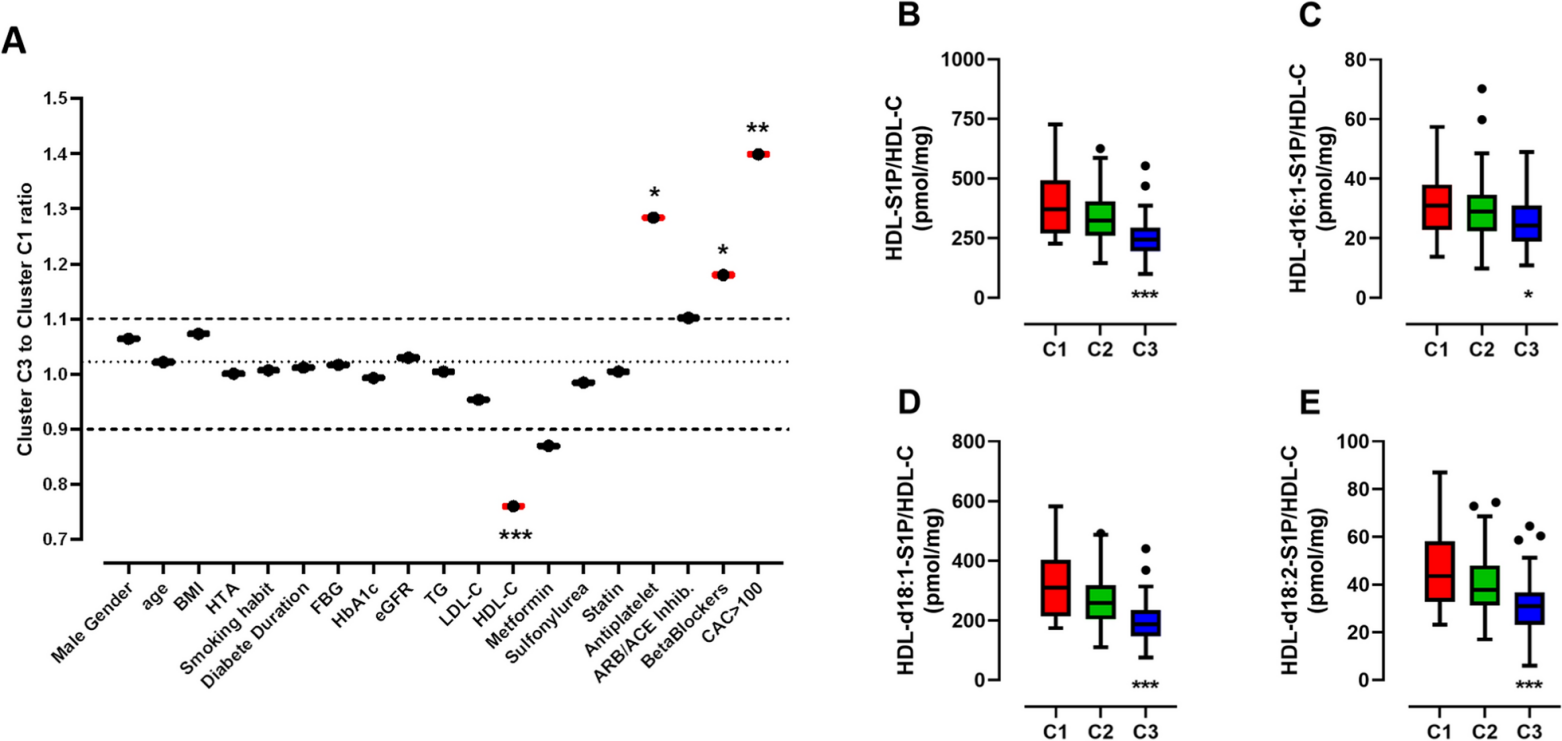
Clinical and biochemical features of type 2 diabetes patients with high-density lipoproteins impoverished in sphingosine-1-phosphate species. Variations of major biological and biochemical parameters among patients with type 2 diabetes belonging to cluster 3 relative to those belonging to cluster 1 panel **A**. HDL-S1P levels expressed relative to HDL-cholesterol levels, total S1P panel **B**, d16:1-S1P (panel **C**), d18:1-S1P panel **D** and d18:2-S1P panel **E**. Cluster 1 (C1, n = 26, red), Cluster 2 (C2, n = 70, green) and Cluster 3, (C3, n = 72, blue). *p < 0.05; **p < 0.005; ***p < 0.0005 Cluster 3 vs Cluster 1

Comparison of major clinical and biochemical characteristics of patients belonging to either cluster C1 or cluster 3, *i.e.* displaying highest or lowest HDL-S1P levels, respectively, revealed that cluster C3 was strongly associated with elevated CAC and reduced circulating HDL-C levels (Fig. 5A). To note, we observed that a higher proportion of patients from cluster 3 was treated with antiplatelet therapy or with beta blockers, consistent with a higher proportion of subjects at high cardiovascular risk in this latter subgroup of patients. In addition, when HDL-S1P levels are expressed relative to HDL-cholesterol levels, the relative enrichment or depletion of HDL particles in S1P species can be estimated. HDL particles isolated from patients belonging to cluster 3 exhibited a reduced total S1P content (Fig. 5B) reflecting reductions in content of individual S1P species, d16:1-S1P (Fig. 5C), d18:1-S1P (Fig. 5D) and d18:2-S1P (Fig. 5E). To note, the observed reduced HDL-S1P content in individuals belonging to Cluster 3 was not associated with insulin resistance (Supplemental Figure S4) or apoM content (Supplemental Figure S5) or the degree of glycation (Supplemental Figure S6). Interestingly, we observed a slight but significant reduction in plasma albumin levels across clusters (Supplemental Figure S7A). Expression of unbound HDL-S1P per mg of plasma albumin, revealed a significant increase of unbound HDL-S1P/albumin across clusters (Supplemental Figure S7B). These data, suggest a potent increase in S1P bound to albumin from Cluster 1 to Cluster 3.

### High-density lipoproteins with a low content of sphingosine-1-phosphate species exhibit impaired anti-inflammatory activities in type 2 diabetes

In order to test whether the variation of the amount of S1P species and precursors in the three clusters of HDL alters their atheroprotective functions, we evaluated anti-inflammatory properties of HDL particles isolated from patients belonging to either cluster C1 or cluster 3, *i.e.* displaying highest or lowest HDL-S1P levels, respectively.

**Fig. 6:**
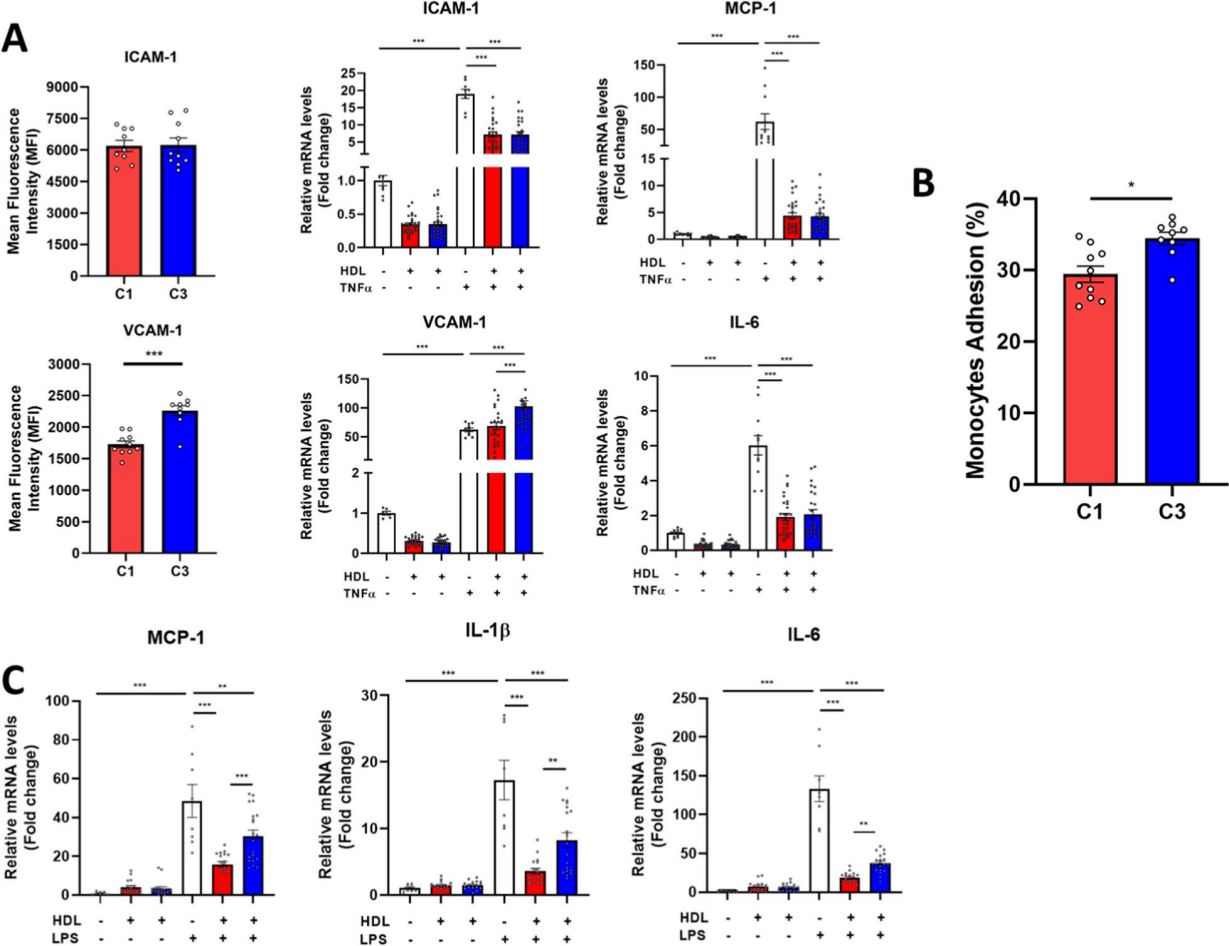
Anti-inflammatory activities of high-density lipoproteins according to the amount of sphingosine-1-phosphate and precursor species. Anti-inflammatory properties of HDL particles isolated patients with type 2 diabetes belonging to cluster 3 (the lowest HDL-S1P; blue bars) relative to those belonging to cluster 1 (the highest HDL-S1P; red bars) evaluated in **A** human umbilical endothelial cells treated or not with TNFα at both cell surface protein (flow cytometry) and mRNA levels (RT-QPCR) and in **C** human THP-1 macrophages treated or not with LPS. **B** Calcein-labelled monocyte-recruitment by endothelial cells was evaluated by flow cytometry and expressed in percentage of FITC-positive cells. Data are presented in means ± SEM. *p < 0.05; **p < 0.005; ***p < 0.0005

To achieve this goal, the impact of HDL-S1P content on the ability of HDL particles to reduce the activation of human vascular endothelial (hUVEC) and the recruitment of monocytes (THP-1) following TNFα treatment to stimulate an acute inflammation was analyzed **(**Fig. 6A, B). We observed higher mRNA levels and cell surface expression of adhesion molecules *VCAM-1* in hUVEC cells incubated in the presence of lowest HDL-S1P from cluster C3 as compared to their counterpart highest HDL-S1P from cluster C1 (Fig. 6A). Interestingly, mRNA levels and cell surface expression of adhesion molecule *ICAM-1* as well as proinflammatory chemokine *MCP-1* and cytokine *IL-6* mRNA levels in hUVEC cells were similar upon incubation with HDL from either cluster C3 or cluster C1, highlighting a specific effect of S1P-enriched HDL on VCAM-1 expression. Additionally, we evaluated the ability of HDL particles to reduce calcein-labelled THP-1 monocyte adhesion on hUVEC cells. In agreement with the higher VCAM-1 expression, an increased number of adherent monocytes at the surface of TNFα-treated endothelial cells was observed in the presence of HDL particles from cluster C3 (the lowest HDL-S1P) as compared to those incubated with HDL from cluster C1 (the highest HDL-S1P) (Fig. 6B).

Finally, to evaluate the anti-inflammatory property of HDL on macrophages, the ability of HDL particles from the two subgroups of patients to inhibit proinflammatory cytokines expression in human THP-1 macrophages following LPS treatment was analyzed (Fig. 6C). We observed a significant increase in *MCP-1*, *IL1β* and *IL-6* mRNA expression in response to incubation of THP-1 macrophages with LPS (Fig. 6C). Interestingly, cells pre-incubated with HDL particles isolated from cluster C1 inhibited the pro-inflammatory action of LPS on all three chemokines and cytokines much more effectively than cells pre-incubated with HDL particles isolated from cluster C3 (Fig. 6C). This indicates that the lowest HDL-S1P particles exhibited a reduced anti-inflammatory action compared to highest HDL-S1P particles. Taken together, these data demonstrate that HDL particles from T2D patients with a low S1P content show a marked overall impairment of their anti-inflammatory properties.

## Discussion

This study demonstrates for the first time that patients with T2D exhibited a reduction of circulating S1P species and their precursors which is associated with a high risk to develop cardiovascular diseases. Furthermore, we demonstrated that cardiovascular risk stratification based on CAC score revealed different patterns of S1P species concentration in HDL of T2D patients. Mechanistically, we propose that such an impoverishment of S1P species in HDL leads to impaired anti-inflammatory properties of HDL which might contribute to atherosclerosis development in T2D.

Coronary artery calcification is a well-established surrogate marker of atherosclerosis and univariate and multivariate analyses presently revealed an inverse relationship between CAC score and plasma levels of S1P in our cohort of patients with T2D. Our findings are consistent with an earlier study showing reduced circulating S1P levels in patients with peripheral artery disease or coronary atherosclerosis [43]. However, S1P levels were also previously proposed as a robust predictor of both occurrence and severity of coronary disease [27] highlighting the complexity of the link between S1P and cardiovascular diseases. This latter is most likely the result of the identity of S1P transporters in the circulation, and more particularly whether S1P species are bound to albumin or lipoproteins [14, 15]. Indeed, the presence of S1P in HDL, the major carrier of S1P in circulation, was largely reported to be associated with multiple atheroprotective biological activities of HDL [13, 16]. Although, no alteration of HDL functions was detected in T2D in comparison to healthy individuals [44], we demonstrated that HDL particles from patients with T2D with a reduced S1P content displayed altered anti-inflammatory properties. Thus, S1P-impoverished HDL exhibited a lower capacity to dampen endothelial activation and monocyte adhesion as well as inflammatory phenotype of macrophages activation which are key steps in the development and progression of early atherosclerotic lesions. In agreement with the ability of HDL to reduce endothelial VCAM-1 expression, atherosclerosis progression and cardiovascular risk [45], we demonstrated that patients with low HDL-S1P levels had an altered capacity to inhibit VCAM-1 expression in human endothelial cells together with a CAC score > 100. Taken together, our findings led us to propose a mechanism through which the reduction of S1P species in HDL would trigger the activation of the vascular endothelium, the recruitment of monocytes and the accumulation of pro-inflammatory macrophages within the arterial intima all contributing to atherosclerosis development in patients with T2D. Importantly, we highlighted that not only the most abundant S1P species, but also minor S1P species previously linked to CVD [29], were also reduced in plasma and HDL from patients with T2D suggesting their potential implication in CAC. Our study reinforces the notion that alteration of lipid composition of HDL is a key event allowing to its dysfunctionality as classically observed in patients with a high CVD risk [46].

It is well recognized that the S1P composition of large HDL is closely linked to the cardioprotective activities of HDL [13, 16]. Interestingly, the loss of large HDL is considered to be a common feature of T2D [47]. In the present study, the extensive analysis of S1P species in both plasma and HDL revealed that, not only the most frequent d18:1-S1P, but all S1P species analyzed were diminished in patients with T2D and a CAC > 100. Clustering of HDL according to their abundance in S1P species and precursors led us to highlight that the low level of all these S1P species in large HDL was a feature of patients with T2D exhibiting dysfunctional HDL and a high CAC. Our study underlies the need to explore the whole diversity of circulating S1P species in patients with an increased CVD risk. In agreement with this, the presence of sphingadienine-based lipid, such as sphingomyelin, a precursor of d18:2-S1P, was associated with an improved antiapoptotic activity of HDL in patient with T2D [47].

It is now well accepted that the biological activities of HDL are closely linked to its size with small, cholesterol-poor HDL exhibiting the highest functionalities, including the anti-inflammatory activity [9, 48]. Interestingly, the function of small HDL was reported to be altered in TD2 [49]. Our findings revealed that sphingosine, a precursor of S1P is reduced in HDL from T2D patients with CAC > 100 and is significantly associated with a lower risk of CAC. Because HDL-sphingosine was not correlated to HDL-C in our cohorts, it suggests that this association results from the sphingosine content of small HDL particles. This original observation reveals that beyond the well-known cardioprotective effect of S1P-HDL, S1P precursors such as sphingosine may also underlie the inverse relationship between HDL and CAC in T2D. Although such a hypothesis is in line with recent studies reporting a role of S1P precursors in the inflammatory response of macrophages [50], this latter needs to be reproduced in larger cohorts of patients with T2D or cardiometabolic diseases.

Several mechanisms underlying a reduction of the S1P content in HDL from patients with T2D have been previously proposed. First of all, the low HDL-C phenotype frequently observed in patients with T2D contributes, in part, to lower plasma HDL-S1P levels. In this context, circulating apoM, the main carrier of S1P in HDL, is reduced in patients with type 2 diabetes as a result of the impact of insulin resistance on the production of ApoM [51]. In consequence, pre-beta HDL formation and further HDL maturation is affected [52] and thus contributes to a decrease in HDL-S1P levels. Second, in the context of high circulating glucose levels as observed during diabetes, the protein and lipid components of HDL particles are highly susceptible to modifications through processes such as non-enzymatic glycation. Specifically, glycation of apoM reduces its ability to bind S1P thus contributing to lower HDL-S1P content [53]. Third, under physiological conditions, circulating S1P is primarily associated with HDL particles [54] while S1P bound to albumin predominates in various pathological situations and has been linked to harmful properties of S1P [55]. Consistent with these latter studies, we presently observed that patients with T2D exhibited elevated features of clinical atherosclerosis characterized by an increase in S1P bound to albumin. In the present study plasma apoM levels were not different between T2D patients with a CAC score below or above 100. Interestingly, a decrease in HDL-S1P, mainly in small HDL, was previously reported by Denimal et al. in patients with type 1 diabetes which was not explained by a decrease in apoM [39]. Moreover, insulin-resistance was not different between the T2D patients with various S1P content in HDL. Therefore, the reduction of S1P content in HDL could equally result from an alteration of the S1P biosynthesis pathway in the liver since circulating concentrations of both S1P species and their precursors were diminished. This latter observation could also indicate that the low S1P level of HDL in patients with T2D with a high CVD risk results from a reduced uptake of circulating S1P species. Such an effect could reflect an alteration of either synthesis or secretion of S1P from key circulating cells such as red blood cells or platelets. Polzin et al. recently revealed that S1P released by platelets exhibited cardioprotection, an effect abolished by the platelet inhibitor cangrelor [56]. Interestingly, patients with T2D having an impoverishment of S1P-HDL and a high CAC score in our study were largely treated with antiplatelet therapy, suggesting a possible side-effect of this treatment in the increased risk of CVD in these patients.

Plaque calcification is a process strongly related to inflammation within the atherosclerotic plaque. Pro-inflammatory macrophages secreting IL-1ß and IL-6 were reported to induce osteoclast differentiation and mineralization of vascular smooth muscle cells, facilitating the calcification of the plaque [57]. Interestingly, the present study provides evidences that HDL particles containing low S1P content exhibited an altered capacity to attenuate the expression of these cytokines in pro-inflammatory macrophages. This supports the idea that the reduced anti-inflammatory activity of S1P-impoverished HDL may contribute to higher calcification of the plaque in T2D.

Alteration of plasma lipidome is a common feature associated with T2D and prediabetes before the apparition of T2D [58], as well as a risk of T2D [59], and may also help to identify new biomarkers of CVD in patients with TD2. In this context, our study provides new important information in the understanding of mechanisms through which alteration of circulating lipidome contributes to CVD in T2D. It identifies HDL-S1P species and their precursors as key players and potential biomarkers of coronary arterial inflammation in patients with T2D with a high CVD risk. New therapeutic strategies in T2D such as glucagon-like peptide-1 (GLP1) receptor agonists were reported to counteract some alterations of circulating lipidome in T2D, including sphingolipids [60, 61], and to reduce the risk of atherosclerotic cardiovascular risk in T2D [62]. Additional studies are therefore needed to investigate if the atheroprotective action of such therapies involves an attenuation of intraplaque due to a restoration of S1P levels.

## Supplementary Information

Supplementary file 1.

## Data Availability

No datasets were generated or analysed during the current study.

## Abbreviations

ACE: Angiotensin converting enzyme
ARB: Angiotensin receptors blockers
BMI: Body mass index
CAC: Coronary artery calcification
CKD: Chronic kidney disease
CV: Cardiovascular disease
DIACART study: DIAbète et calcification ARTerielle
HbA1c: Hemoglobin A1c
HDL: High-density lipoprotein
HDL-C: High-density lipoprotein-cholesterol
HOMA-IR: Homeostasis model assessment of insulin resistance index
ICAM-1: InterCellular adhesion molecule 1
IL-1ß: Interleukin 1 beta
IL-6: Interleukin 6
LC–MS/MS: Liquid chromatography coupled with tandem mass spectrometry
LDL-C: Low-density lipoprotein-cholesterol
NO: Nitric oxide
PCA: Principal component analysis
PMA: Phorbol 12-myristate 13-acetate
S1P: Sphingosine 1-phosphate
SphK: Sphingosine kinase
SPT: Serine palmitoyl transferase
T2D: Type 2 diabetes
TNFα: Tumor necrosis factor alpha
VCAM-1: Vascular cell adhesion protein 1
ICAM-1: InterCellular adhesion molecule 1
