# Supplementary material for: Inverse relationship between circulating sphingosine-1-phosphate and precursor species and coronary artery calcification score in type 2 diabetes
Source: Cardiovasc Diabetol. 2025 Feb 21;24:85. doi: 10.1186/s12933-025-02624-9 (PMC11846453; doi:10.1186/s12933-025-02624-9)

Supplemental

Inverse relationship between circulating sphingosine species and coronary artery calcification score in type 2 diabetes

Wilfried LE GOFF^1^**^†^**, Olivier BOURRON^1,2^**^†^**, Clément MATERNE^1^, Sophie GALIER^1^, Franck PHAN^1,2^, Sophie TAN-CHEN^6^, Isabelle GUILLAS^1^, Agnès HARTEMANN^3^, Joe-Elie SALEM^3^, Alban REDHEUIL^4^, Fabienne FOUFELLE^1^, Hervé LE STUNFF^5^**^*^**, Eric HAJDUCH^1^**^*^**, Maryse GUERIN^1^**^*^**

1. Sorbonne Université, INSERM, Foundation for Innovation in Cardiometabolism and Nutrition (ICAN), UMR_S1166, F-75013 Paris, France.

2. Assistance Publique-Hôpitaux de Paris (APHP), Diabetology Department, La Pitié Salpêtrière-Charles Foix University Hospital, Paris, France.

3.Sorbonne Université, APHP, INSERM, CIC-1901, Hopital Pitié-Salpétrière, Paris, France

4. Laboratoire d'Imagerie Biomédicale INSERM_1146, CNRS_7371, Assistance Publique‑Hôpitaux de Paris (APHP), ICT Cardiovascular and Thoracic Imaging Unit, Pitié Salpêtrière University Hospital, Paris, France.

5. Université Paris-Saclay, CNRS UMR 9197, Institut des Neurosciences Paris-Saclay, Saclay, France.

6. Centre de Recherche des Cordeliers, INSERM, Sorbonne Université, Paris, France.

**^†^**Wilfried Le Goff and Olivier Bourron contributed equally to this work as co-first authors.

**^*^** Hervé Le Stunff, Eric Hajduch and Maryse Guerin contributed equally to the manuscript and must be considered as joint last authors.

**Correspondence:**

**Maryse GUERIN, PhD. Eric Hajduch, Ph.D Hervé Le Stunff, Ph.D.**

INSERM UMRS_1166 INSERM UMRS_1166 Institut des Neurosciences Paris-Saclay

Faculté de Santé-Pitié Salpetrière Faculté de Santé-Pitié Salpetrière CNRS UMR 9197

91, boulevard de l’Hôpital 91, boulevard de l’Hôpital Université Paris-Saclay

75013 Paris 75013 Paris Paris-Saclay

France France France

email: [maryse.guerin@inserm.fr](mailto:maryse.guerin@inserm.fr) email: [eric.hajduch@sorbonne-universite.fr](mailto:eric.hajduch@sorbonne-universite.fr) hlestunff62@gmail.com

**Supplemental Table S1: Human Primers for quantitative RT-PCR**

| **Gene** | **Forward** | **Reverse** |
| --- | --- | --- |
| *NONO* | aggaaggattcaagggaacc | gcatggcacctctgttgtt |
| *TUBA* | gatgctgccaataactatgcccgag | gaaaaccaagaagccctgaagacgg |
| *HSP90AB1* | agcctacgttgctcactattacg | gaaaggcaaaagtctccacct |
| *IL-1ß* | tccagggacaggatatggag | tcatctttcaacacgcagga |
| *IL-6* | caggagcccagctatgaact | agcaggcaacaccaggag |
| *VCAM-1* | gattctgtgcccacagtaaggc | tggtcacagagccaccttcttg |
| *ICAM-1* | agcggctgacgtgtgcagtaat | tctgagacctctggcttcgtca |
| *MCP-1* | gaccacctggacaagcaaac | taaaacagggtgtctgggga |

| Target anitgen | Species reactivity | Source | Reference # | Working solution | Dye | Lot # | Persistent ID/URL |
| --- | --- | --- | --- | --- | --- | --- | --- |
| Fixable viability dye | N/A | eBiosciences | 65-0867-18 | 1/1000 | eF520 | N/A | https://www.thermofisher.com/order/catalog/product/65-0867-18 |
| VCAM-1/CD106 | Human | BD Bioscience | 555647 | 1/5 | PE | 51-10C9 (RUO) | https://www.bdbiosciences.com/en-fr/products/reagents/flow-cytometry-reagents/research-reagents/single-color-antibodies-ruo/pe-mouse-anti-human-cd106.555647 |
| ICAM-1/CD54 | Human | BD Bioscience | 559771 | 1/5 | APC | HA58 (RUO) | https://www.bdbiosciences.com/en-fr/products/reagents/flow-cytometry-reagents/research-reagents/single-color-antibodies-ruo/apc-mouse-anti-human-cd54.559771 |
| Calcein-AM | N/A | ThermoFisher Scientific | C3100MP |  | FITC equivalent | N/A | https://www.thermofisher.com/order/catalog/product/C3100MP |
| FcR Blocker | Human | Miltenyi Biotec | 130-059-901 | 1/100 | N/A | N/A | https://www.miltenyibiotec.com/FR-en/products/fcr-blocking-reagent-human.html |
| eBioscience™ Foxp3 / Transcription Factor Fixation | N/A | Invitrogen | 00-5521-00 | N/A | N/A | N/A | https://www.thermofisher.com/order/catalog/product/00-5521-00?SID=srch-srp-00-5521-00 |

**Supplemental Table S2: Cell markers for FACS analysis and cell sorting.**

| Product | Source | Reference # | Persistent ID/URL |
| --- | --- | --- | --- |
| RPMI-1640 | Sigma | R0883 | https://www.sigmaaldrich.com/FR/fr/product/sigma/r0883?srsltid=AfmBOoqYCJQCQIR-rPG_G9rePRXO7mqJ7Fa3zB0wqUfUJeXR3tvfAIei |
| Endothelial cells growth media 2 | Promocell | C-22111 | https://promocell.com/fr_fr/endothelial-cell-growth-medium-2.html |
| Fœtal Bovine Serum | Sigma | F2442 | https://www.sigmaaldrich.com/FR/fr/product/sigma/f2442 |
| Trypsin-EDTA | Sigma | T4049 | https://www.sigmaaldrich.com/FR/fr/product/sigma/t4049 |
| TrypLE | ThermoFisher Scientific | 12605010 | https://www.thermofisher.com/order/catalog/product/12605010 |
| PMA | Sigma | P8139 | https://www.sigmaaldrich.com/FR/fr/product/sigma/p8139?utm_source=google&utm_medium=cpc&utm_campaign=20847908568&utm_content=158253545722&gclid=Cj0KCQjwiOy1BhDCARIsADGvQnCIf4kguqwrtnDXmdFVJzJe97jjW3CyGnmv4NnhZxquEMnh4-f_JgYaAmfGEALw_wcB |
| TNF-α | Sigma | SRP3177 | https://www.sigmaaldrich.com/FR/fr/product/sigma/srp3177 |
| LPS | Sigma | L2630 | https://www.sigmaaldrich.com/FR/fr/product/sigma/l2630 |

**Supplemental Table S3. Major resources table**

| Variables | OR [95%CI] | p, value |
| --- | --- | --- |
| Age, year | 2.52 [2.51-5.30] | <0.0001 |
| Male Gender | 2.23 [1.08-4.53] | 0.0283 |
| BMI, kg/m² | 1.07 [0.78-1.50] | 0.67 |
| HTA | 3.43 [1.63-7.21] | 0.0011 |
| Smoking habit | 2.04 [1.06-7.21] | 0.0318 |
| FBG, mmol/l | 0.81 [0.59-1.11] | 0.19 |
| HbA1c | 0.82 [0.60-1.12] | 0.21 |
| CKD | 1.96 [1.02-3.82] | 0.0227 |
| Triglycerides | 1.43 [0.96-2.31] | 0.11 |
| Total Cholesterol | 0.65 [0.47-0.89] | 0.0086 |
| LDL-C | 0.52 [0.36-0.73] | 0.0003 |
| HDL-C | 0.82 [0.60-1.13] | 0.21 |
| Metformin | 0.83 [0.35-1.84] | 0.67 |
| Sulfonylurea | 1.07 [0.56-2.05] | 0.84 |
| DPP4 Inhibitor | 0.97 [0.48-2.04] | 0.94 |
| αglucosidase inhibitor | 0.33 [0.01-8.47] | 0.43 |
| Glinide | 0.32 [0.06-1.79] | 0.17 |
| GLP1 receptor agonist | 0.31 [0.09- 1.03] | 0.051 |
| Insulin | 1.29 [0.68-2.47] | 0.44 |
| Statin | 4.28 [1.96-9.45] | 0.0003 |
| Ezetimibe | 1.68 [0.59-6.02] | 0.37 |
| Fibrates | 1.18 [0.27-8.12] | 0.84 |
| Antiplatelet | 11.6 [5.65-25.1] | <0.0001 |
| ARB and ACE inhibitors | 3.52 [1.77-7.05] | 0.0003 |
| Beta Blockers | 5.73 [2.83-12.3] | <0.0001 |

**Supplemental Table S4: Univariate analysis of the relationship between variables and Coronary Artery Calcification score**


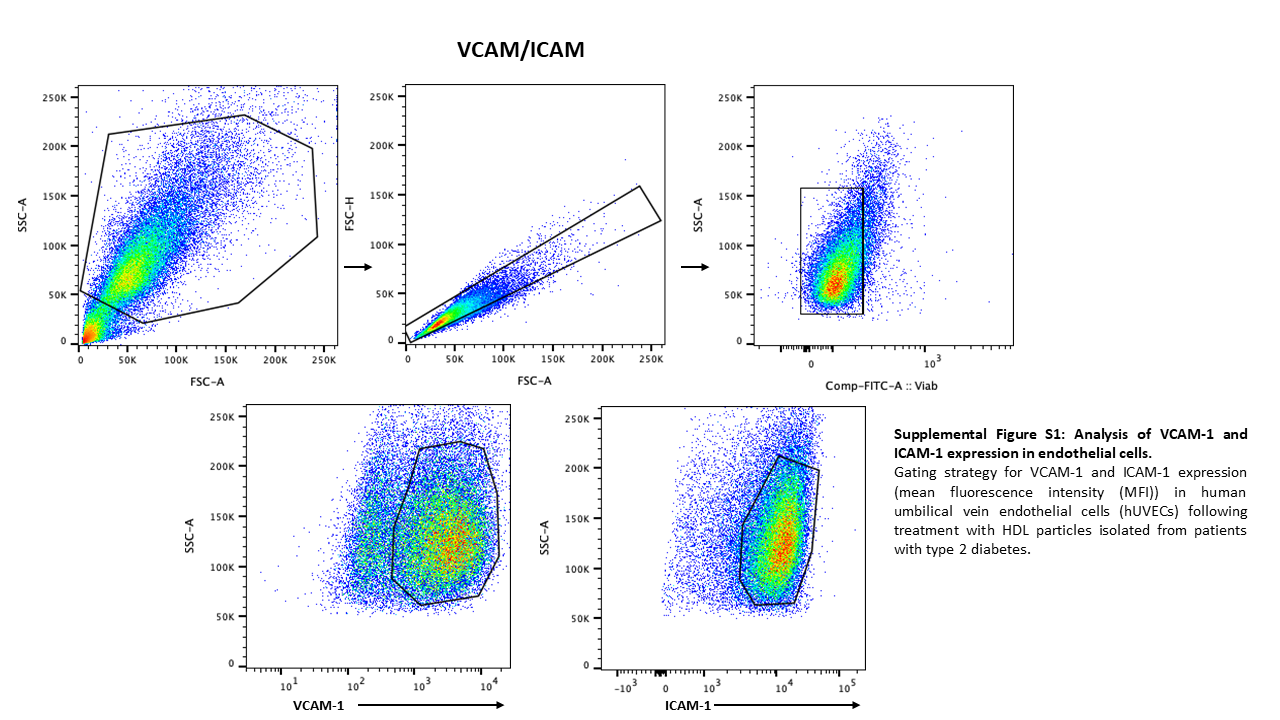


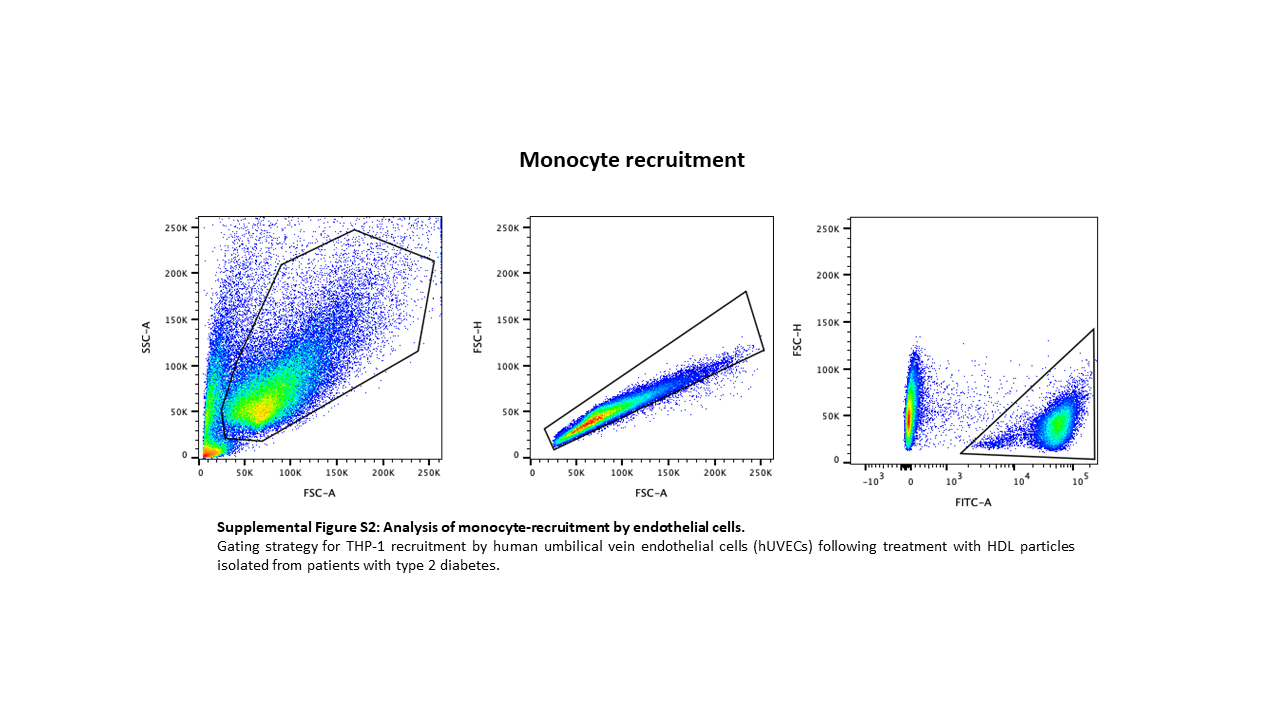


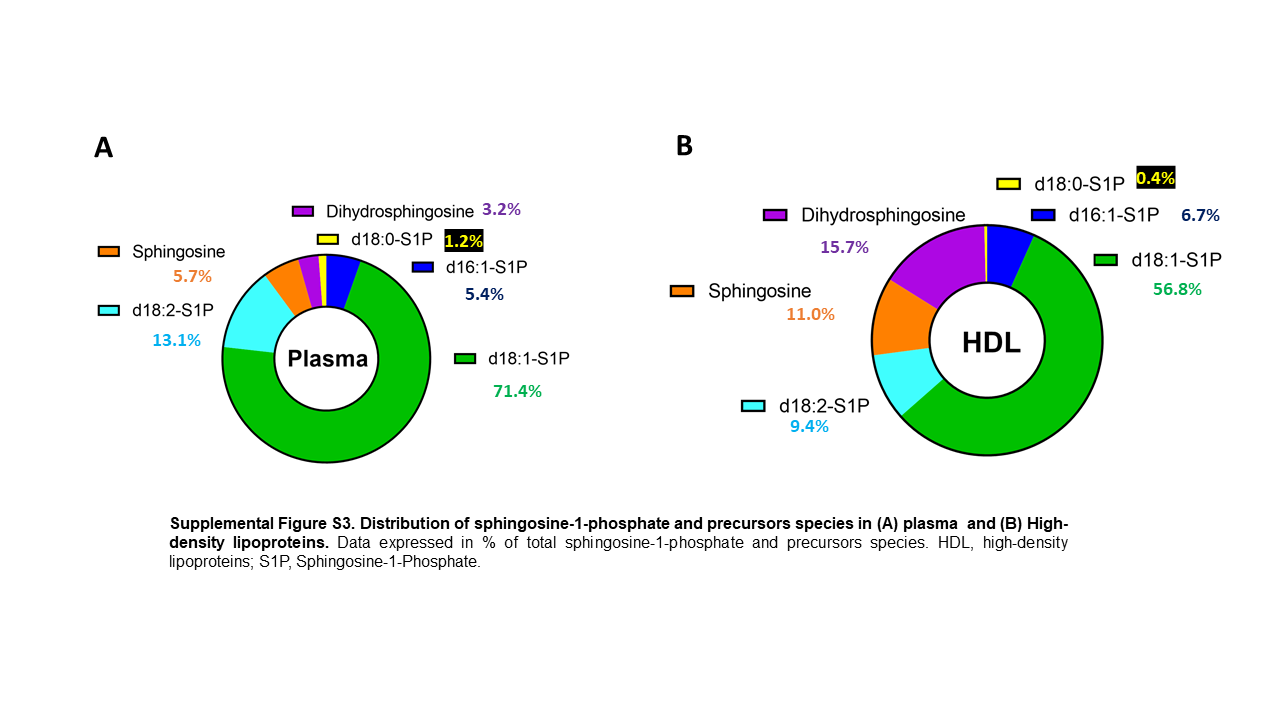


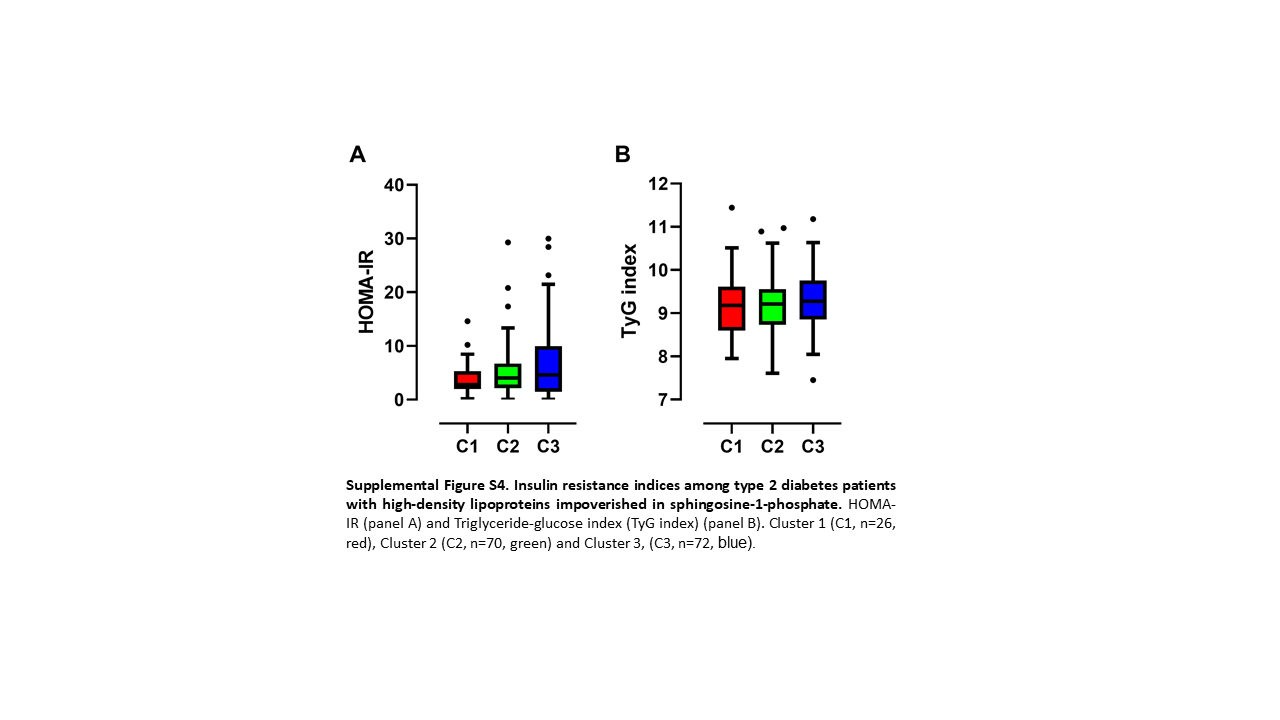


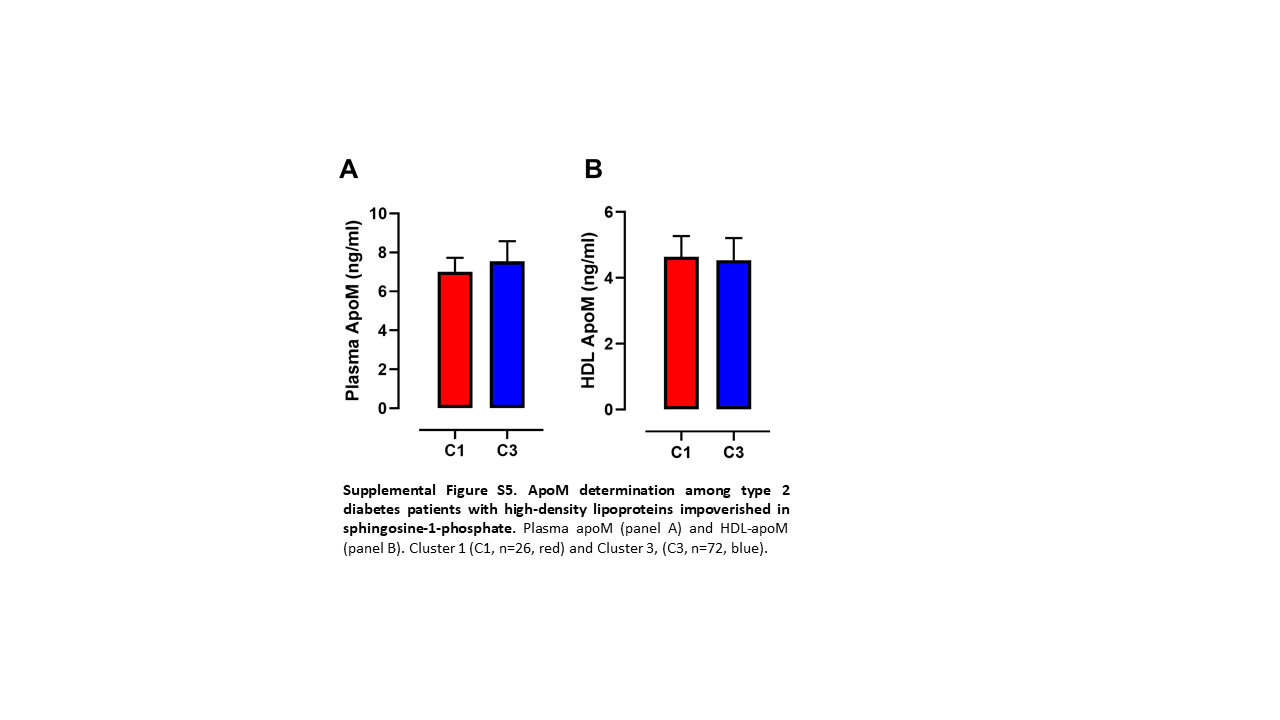


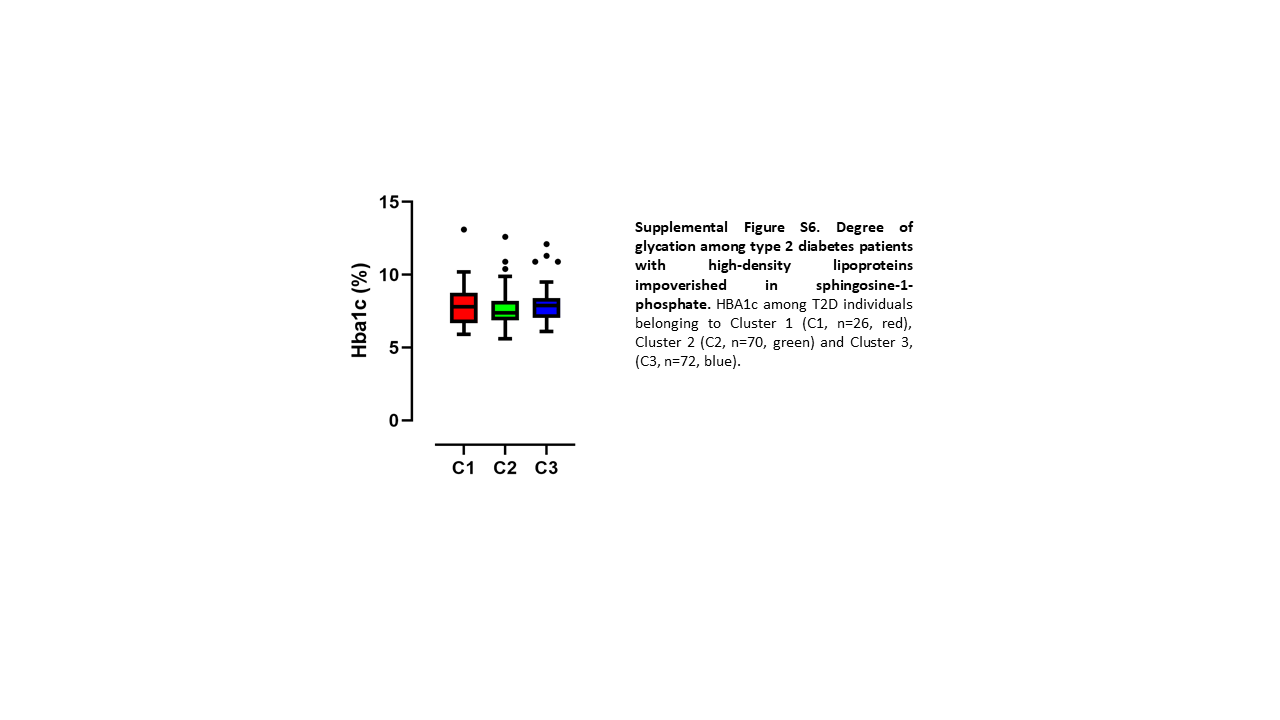


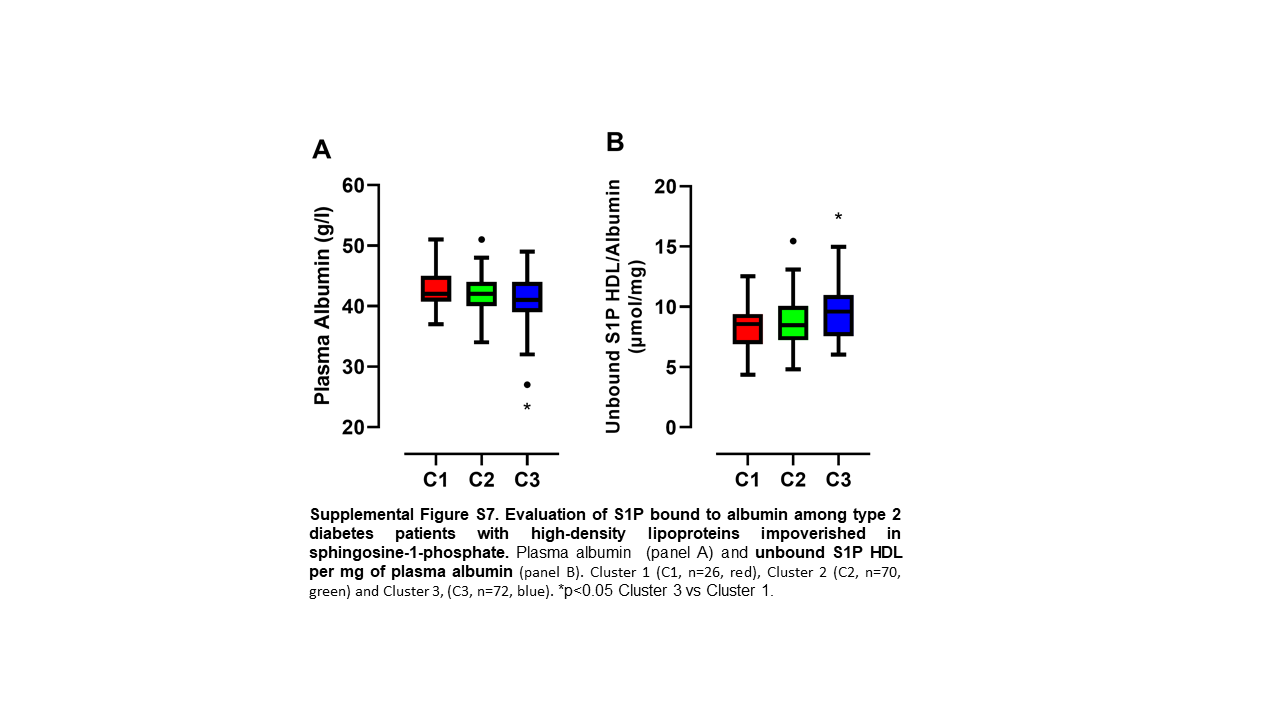

Supplement: Supplementary file 1 [file 12933_2025_2624_MOESM1_ESM.docx]
